# A New Zealand Perspective on the Application and Regulation of Gene Editing

**DOI:** 10.3389/fpls.2018.01323

**Published:** 2018-09-12

**Authors:** Steffi Fritsche, Charleson Poovaiah, Elspeth MacRae, Glenn Thorlby

**Affiliations:** Scion, Rotorua, New Zealand

**Keywords:** gene editing, New Zealand, regulation, traits, industry

## Abstract

New Zealand (NZ) is a small country with an export-led economy with above 90% of primary production exported. Plant-based primary commodities derived from the pastoral, horticultural and forestry sectors account for around half of the export earnings. Productivity is characterized by a history of innovation and the early adoption of advanced technologies. Gene editing has the potential to revolutionize breeding programmes, particularly in NZ. Here, perennials such as tree crops and forestry species are key components of the primary production value chain but are challenging for conventional breeding and only recently domesticated. Uncertainty over the global regulatory status of gene editing products is a barrier to invest in and apply editing techniques in plant breeding. NZs major trading partners including Europe, Asia and Australia are currently evaluating the regulatory status of these technologies and have not made definitive decisions. NZ is one of the few countries where the regulatory status of gene editing has been clarified. In 2014, the NZ Environmental Protection Authority ruled that plants produced via gene editing methods, where no foreign DNA remained in the edited plant, would not be regulated as GMOs. However, following a challenge in the High Court, this decision was overturned such that NZ currently controls all products of gene editing as GMOs. Here, we illustrate the potential benefits of integrating gene editing into plant breeding programmes using targets and traits with application in NZ. The regulatory process which led to gene editing's current GMO classification in NZ is described and the importance of globally harmonized regulations, particularly to small export-driven nations is discussed.

## Introduction

Primary exports are critical to New Zealand's (NZ's) economy providing both employment and export revenue. In 2017, this totalled NZ$38 billion of which the dairy industry contributed NZ$ 14.6 billion, red meat and wool NZ$8.4 billion, forestry NZ$5.5 billion and horticulture NZ$5.1 billion (Ministry for Primary Industries, 2018b). New Zealand's pasture-based dairy industry is the world's largest dairy exporter and accounts for a third of the world's dairy trade (Chobtang et al., 2017a). Sheep and beef make up the majority of animal-based exports but venison and wool are significant contributors. The NZ sheep and beef sector exports close to 90% of its production. Forestry, based around exotic plantation forests (primarily radiata pine and Douglas-fir), covers 1.751 million hectares—approximately 7% of NZ's land area (Ministry for Primary Industries, 2018a). The horticultural sector is predominately fruit based and led by kiwifruit, of which 95% of production is exported, wine, apple and pear are also exported in significant volumes. The main destinations for primary exports are China (NZ$9.1 billion), Australia (NZ$4.3 billion), and the US (NZ$4.0 billion), with Japan, South Korea and Europe also being significant markets.

Nations with small domestic markets like NZ face pressure to continuously adjust and innovate in order to maintain global competitiveness (Vitalis, 2007). To support this, NZ has a long history of implementation of agritech innovation (Easton, 1997; Vitalis, 2007; Hedley, 2015) including the use of genetic technologies (Harris et al., 2009; Kumar et al., 2012). In order to maintain NZ's position whilst providing sustainable solutions to the challenges of global food security and climate change a step change in productivity beyond that which has been possible through conventional breeding will be required (Williams et al., 2007). Solutions are also urgently required for the increased threat from pests and diseases. In the last decade the kiwifruit and forestry industry have suffered considerable losses from emerging diseases (Vanneste, 2012; Scott and Williams, 2014). Myrtle rust, which has caused worldwide damage to both agricultural and native ecosystems, arrived in NZ in 2017 (Office of the Minister of Conservation, 2017). Biotechnology-based improvements have the potential to be an important tool in delivering this. The unprecedented uptake of genetically modified (GM) crops over the last 20 years, such that 189.8 million hectares of GM crops were planted in 24 countries in 2017 (ISAAA, 2017) is testimony to this. GM crops are now cultivated on more than 10% of the worlds farmland and comprise 80% of global cotton and 77% of soybean plantings (ISAAA, 2017; Taheri et al., 2017).

Currently no GM crops are grown in NZ. The globally traded cash crops (corn, soybean, canola and cotton) that make up the majority of current GM plantings are not widely grown and do not provide a compelling value proposition for NZ. In contrast, NZ aims to supply high value innovative products that are not cultivated on a global large scale e.g., kiwifruit and radiata pine. The time and cost of developing and gaining regulatory approval for GM versions of these for the NZ market is prohibitive. The lack of relevant GM crops has meant that there has not been recent nationwide debate on the merits of these technologies in NZ (Bryan and Roberts, 2015).

Over the last decade genome editing methods based on Zinc finger nucleases (Urnov et al., 2010), TALENs (Chen and Gao, 2013), CRISPR/Cas (Doudna and Charpentier, 2014) systems have rapidly revolutionized both basic and applied biology. The wide-ranging applications of this technology have been extensively reviewed elsewhere (Voytas, 2013; Carroll, 2014; Wang et al., 2016a; Brooks and Gaj, 2018). In this review, we will focus on the use of gene editing to carry out targeted mutagenesis on plant species where no DNA template is used. We believe this technology has the ability to encourage a paradigm shift in the incorporation of biotechnology into NZ plant breeding programmes. Particularly if, as seems likely, it is ultimately regulated in a less burdensome way than GM technology. Here, we give examples of the traits that could be modified to give NZ relevant outcomes, describe the current regulatory landscape, and discuss the implications of this on the future innovation in NZ plant-based primary industries.

## Potential applications of gene editing in New Zealand

Gene editing offers the potential to produce a step change in NZ primary industry productivity, biosecurity and speed of innovation. This is particularly the case for perennial crops with slow or complex breeding cycles that are a feature of NZ's plant-based exports. Although gene editing has already been demonstrated for a number of NZ relevant crops (Table 1), it is still to be implemented for a number of important species particularly conifer forestry species. This review focuses on plant-based applications, however, uses in animal breeding (Wei et al., 2018) and control of introduced pests via gene drive technology (Dearden et al., 2018) are also in development. Below, as examples, we describe possible scenarios where plant-based gene editing could have an impact on primary production and innovation.

**Table 1:** Examples of species relevant to New Zealand's plant-based primary industries that have been modified using genome editing technologies.

**Species**	**Method**	**Tissue**	**Purpose**	**Result**	**References**
**WOODY SPECIES**					
Apple	CRISPR/Cas9 RNPs	Protoplasts	Mutate *DIPM-1, DIPM-2*, and *DIPM-4* to increase resistance to fire blight disease.	No plants regenerated	Malnoy et al., 2016
Apple	CRISPR/Cas9	Agrobacterium-mediated transformation of leaf disks	Mutate *phytoene desaturase (PDS)* gene	First generation albino plants regenerated with mutated PDS gene	Nishitani et al., 2016
Apple	ZFN	Agrobacterium-mediated transformation of leaf disks	Activation of a mutated *UidA* gene	First generation plants expressing GUS regenerated	Peer et al., 2015
Grape	CRISPR/Cas9	Proembryonal mass	Increased resistance to *Botrytis cinerea*	First generation plants regenerated with increased resistance to *Botrytis cinerea*	Wang et al., 2018a
Kiwifruit	CRISPR/Cas9	Agrobacterium-mediated transformation of leaf disks	Mutate *phytoene desaturase (PDS)* gene	First generation albino plants regenerated with mutated PDS gene	Wang et al., 2018b
Sweet Orange	CRISPR/Cas9	Agroinfiltration of leaf	Mutate *phytoene desaturase (PDS)* gene	No plants regenerated	Jia and Wang, 2014
Poplar	ZFN	Agrobacterium-mediated transformation of leaf disks	Mutate *LEAFY and AGAMOUS* orthologs in poplar	First generation plants regenerated	Lu et al., 2016
Poplar	CRISPR/Cas9	Agrobacterium-mediated transformation of leaf disks	Mutate *phytoene desaturase (PDS)* gene	First generation albino plants regenerated	Fan et al., 2015
Poplar	CRISPR/Cas9	Agrobacterium-mediated transformation of leaf disks	Mutate *4-coumarate:CoA ligase* (*4CL*) gene	First generation plants regenerated with decreased lignin	Zhou et al., 2015
**FORAGE CROPS**					
Alfalfa	CRISPR/Cas9	Agrobacterium-mediated transformation of leaf disks	Mutate *squamosa promoter binding protein like 9 (SPL9)* gene	First generation plants regenerated	Gao et al., 2018
**VEGETABLE CROPS**					
Tomato	CRISPR/Cas9	Agrobacterium-mediated transformation of cotyledon segments	Mutate tomato *ARGONAUT7* (*SlAGO7*)	First and second generation plants were produced	Brooks et al., 2014
Tomato	CRISPR/Cas9	Agrobacterium-mediated transformation of cotyledon segments	Mutate *SlIAA9, SLAGL6*	Parthenocarpy	Klap et al., 2017; Ueta et al., 2017
Potato	CRISPR/Cas9	Agrobacterium-mediated transformation of stem segments	Mutate *StIAA2* encoding an Aux/IAA protein	First generation plants regenerated	Wang et al., 2016b
Potato	Geminivirus mediated CRISPR/Cas9	Agrobacterium-mediated transformation of stem segments	Mutate *ACETOLACTATE SYNTHASE1* (*StALS1*)	Herbicide tolerant plant generated	Butler et al., 2016
Cucumber	CRISPR/Cas9	Agrobacterium-mediated transformation of cotyledon segments	Mutate *eIF4E* to develop resistance to virus	Plants exhibited immunity to Cucumber vein yellowing virus (Ipomovirus) infection and resistance to the Zucchini yellow mosaic virus and Papaya ring spot mosaic virus-W	Chandrasekaran et al., 2016
Lettuce	CRISPR/Cas9 RNPs	Protoplasts	Mutate *BRASSINOSTEROID INSENSITIVE 2* (*BIN2*) gene	Whole plants with mutated *BIN2* regenerated from protoplasts	Woo et al., 2015

### Control of invasive conifers by manipulation of reproduction

NZ faces serious ecological, economic and cultural challenges from invasive tree species that have “escaped” by seed dispersal from planted forests and shelter belts (Richardson and Rejmánek, 2004). Several exotic conifer species that have become established outside plantations now occupy ~1.8 million ha of land, and are expanding by 6% annually (Froude, 2011). The government has declared these to be the most significant weed problem facing NZ (The New Zealand Government, 2016a) with control of the existing population costing an estimated NZ$15 million each year. The social and economic costs of these escapes is challenging the ability of forest owners to carry out new plantings with commercially advantageous, but potentially invasive species such as Douglas-fir. The capability to generate trees that are unable to reproduce would allow control programs to focus on the existing populations and give freedom to operate for new plantings. Prevention of cone development is also predicted to increase growth and wood development by the redirection of energy and nutrients toward vegetative growth (Santos-del-Blanco and Climent, 2014).

Gene editing provides an attractive approach to prevent the generation of new escapees via targeted mutagenesis of genes essential for normal sexual reproduction. Genes involved in the transition from the juvenile to reproductive growth phase, cone initiation or development, and pollen formation and development are potential targets (Strauss et al., 1995). If transgene-free edited trees are required, DNA-free delivery methods would be necessary because the long breeding cycles of conifers would prevent timely segregation of transgenes from edited genes.

### Rapid breeding in apple

Breeding of new apple varieties is a slow process limited by a long-lasting juvenile stage taking more than two decades to bring a new variety into the market (Flachowsky et al., 2009). Shortening the juvenile stage has been the subject of intensive research and is a major objective in breeding (Meilan, 1997). Early flowering has been demonstrated in apple through the overexpression of beech *MADS4* and Arabidopsis *FT* gene (Flachowsky et al., 2007; Yamagishi et al., 2011). This technology has been used to rapidly breed fire blight resistance into apple within 7 years (Schlathölter et al., 2018). A similar result has been obtained using antisense-based silencing of *MdTFL1* expression (Kotoda et al., 2006). Gene editing could be used to knock out the expression of *MdTFL1* to reproduce this early flowering phenotype. This would allow rapid breeding of new cultivars through several cycles after which the edited gene could be crossed out to restore the non-engineered flowering phenotype without any trace of the modification.

### Improved pasture quality

The dairy, meat and wool industries in NZ draw a significant market advantage from the predominantly pasture-based feed. Limiting environmental impacts whilst meeting the increase in global demand for dairy products requires improvements in pasture productivity (Chobtang et al., 2017b). Forage pastures generally consist of ryegrass, alfalfa and clover. Of these, annual and perennial ryegrass are most common. Gene editing provides tools to improve productivity and reduce disease either through the direct manipulation of forage crops or via manipulation of endophytes. The incorporation of herbicide tolerance (Butler et al., 2016) and easier digestibility (Li et al., 2018) have both been successfully introduced into plants by gene editing and research to increase energy values is underway. These are likely to offer routes to both increased productivity and a reduced environmental footprint.

Forage grasses like ryegrass are usually infected with symbiotic fungal endophytes (Latch et al., 1984) which produce secondary metabolites that protect the plant from invertebrate pests (Mortimer and Di Menna, 1983), give higher growth rates, tolerance to abiotic stress (West and Gwinn, 1993), and produce more dry matter than non-infected plants (Popay et al., 1999). These benefits can be compromised by the production of high levels of indole-diterpenes and alkaloids that have negative impacts on livestock e.g., ryegrass staggers in sheep (Fletcher and Harvey, 1981; Thom et al., 2007). To minimize the toxicity of these symbionts, strains of endophytes were selected that produced low levels of these alkaloids and indole-diterpenes (Davies et al., 1993). Molecular analysis revealed these lower levels were due to deletions within the coding sequence of genes in the biosynthetic pathway (Young et al., 2009). Gene editing will allow the modification of biosynthetic pathways to decrease or eliminate toxins and increase the production of desirable metabolites without the need to screen for extremely rare natural variants.

## Regulation of gene editing in New Zealand

The global social and regulatory landscape surrounding GM crops remains complex with many different regulatory systems in place (Wolt et al., 2016; Davison and Ammann, 2017). The primary difference being whether a process or product driven framework is used (Ishii and Araki, 2017). As yet there is not a global consensus on the regulation of gene editing which was developed after current regulatory frameworks were put in place. Several nations, including the USA, Canada and Argentina, have decided that gene editing technologies where the final plant does not contain introduced DNA will not be regulated (Whelan and Lema, 2015; Ishii and Araki, 2017; Waltz, 2018). In contrast the European Union recently decided that all gene editing technologies will be regulated in the same way as conventional GM organisms (Callaway, 2018; Kupferschmidt, 2018). Others, including the two main destinations for NZ's primary exports, China and Australia, are yet to decide on their regulatory approach.

New Zealand regulates GM organisms using a stringent process driven regulatory framework—the Hazardous Substances and New Organisms (HSNO) Act 1996. The Act defines a GMO very broadly as any organism where the genes or genetic material have been modified by *in vitro* techniques (Table 2a). A number of technologies that were in use at the time the Act was passed are captured by this broad definition e.g., somaclonal variation, cell fusion, and chemical and physical mutagenesis. To counter this, a number of technologies that meet the definition of generating a GMO are excluded from being regulated by the HSNO (Organisms Not Genetically Modified) Regulations 1998 (Table 2b).

**Table 2:** The regulation of GMOs and gene editing in New Zealand.

(a) HSNO Act – Definition of GMO	**Section 2** Genetically modified organism means, unless expressly provided otherwise by regulations, any organism in which any of the genes or other genetic material- (a) have been modified by in vitro* techniques; or (b) are inherited or otherwise derived, through any number of replications, from any genes or other genetic material which has been modified by *in vitro* techniques (* the term *in vitro* is not defined by the Act)
(b) HSNO (Organisms Not Genetically Modified) Regulations 1998	**3 Organisms not genetically modified** (1) For the purposes of the Act, the following organisms are not to be regarded as genetically modified: (a) organisms that result solely from selection or natural regeneration, hand pollination, or other managed, controlled pollination; (b) organisms that are regenerated from organs, tissues, or cell culture, including those produced through selection and propagation of somaclonal variants, embryo rescue, and cell fusion (including protoplast fusion or chemical or radiation treatments that cause changes in chromosome number or cause chromosome rearrangements); (c) organisms that result solely from artificial insemination, superovulation, embryo transfer, or embryo splitting; (d) organisms modified solely by (i) the movement of nucleic acids using physiological processes, including conjugation, transduction, and transformation; and (ii) plasmid loss or spontaneous deletion; (e) organisms resulting from spontaneous deletions, rearrangements, and amplifications within a single genome, including its extrachromosomal elements.
(c) HSNO (Organisms Not Genetically Modified) Amendment to Regulations - 29 September 2016	**Regulation clause 3(1)(b)** (1) For the purposes of the Act, the following organisms are not to be regarded as genetically modified: (b) organisms that are regenerated from organs, tissues, or cell culture, including those produced through selection and propagation of somaclonal variants, embryo rescue, and cell fusion including protoplast fusion: (ba) organisms that result from mutagenesis that uses chemical or radiation treatments that were in use on or before 29 July 1998

*The HSNO Act definition of a GMO (a), and regulations excluding certain technologies from being regulated in the original (b) and revised (c) regulations are given. The unorthodox use of the word including at the beginning of the list of except techniques in section (b) is underlined*.

### Application to determine status of gene editing

The HSNO Act, under section 26, provides a mechanism for an applicant to ask for a determination by the Environmental Protection Authority (EPA) as to whether, an organism is regulated as a GM in NZ (Kershen, 2015). In 2012, Scion, a forestry-focused Crown Research Institute, used this procedure to seek a determination on how gene edited organisms would be regulated. The HSNO definition (Table 2a) includes a clause specifying that genetic modifications “inherited or otherwise derived, through any number of replications” would be classed as GMOs. Scion's application, which was submitted before CRISPR/Cas9 technology was developed, thus sought to determine “*whether the use of custom Zinc Finger Nucleases and custom Transcription Activator-Like Effectors results in organisms classed as genetically modified organisms*” when the editing complex was delivered without the use of a transgene to carry the editing machinery.

Scion's application argued that gene editing technologies that did not include the insertion of a transgene into host genome were similar in process and outcome to chemical mutagenesis. As such they should be included within the HSNO regulations exception of “*chemical or radiation treatments that cause changes in chromosome number or cause chromosome rearrangements*” (Table 2b). Scion noted that the list of techniques that were excluded from regulation was preceded by the word included (underlined for emphasis in Table 2b) suggesting that these were example techniques and not a closed list.

### EPA decision

In their decision of April 2013, the EPA concluded that the non-transgenic gene editing approach proposed by Scion had similarities to both chemical mutagenesis and genetic manipulation. However, because the changes involved the use of a chemical agent (in this case, a protein) without the introduction of foreign DNA it is more similar to chemical mutagenesis (Environmental Protection Authority, 2013). The EPA further stated that the Regulations (Table 2b) exclude products of chemical mutagenesis from being regulated as GMOs under the Act and that the proposed modifications were sufficiently similar to those listed in the Regulations and should also be excluded, and organisms arising from them should not be considered GMOs.

### High court challenge

The EPA decision was appealed by the Sustainability Council of New Zealand in the High Court and the case was heard in November 2013. The key consideration of the judgement, issued on the 20th May 2014, was “*whether the specific techniques (listed in Table* *2b**) are a closed list of techniques that are exempted, or whether they describe a category of the kind of techniques that are excepted (so that other techniques which are sufficiently similar to those techniques are also exempted)*” (The High Court of New Zealand, 2014). The Court concluded that the list of techniques listed in the HSNO (Organisms Not Genetically Modified) Regulations 1998 (Table 2b) are a closed list and that adding to the exceptions list is a political decision and not an administrative decision (Kershen, 2015). On this basis the EPA's original decision was quashed and all gene editing is currently regulated as a GM procedure in NZ.

### Implications of the decision

In the court ruling the judge pointed out that the regulations are not well drafted, brackets are in the wrong place and the grammar poor. This reinforced her interpretation that the unorthodox use of the word “including” before start of the list of techniques that do not produce GMOs (Table 2b) does not constitute a list of examples but rather a closed list. She also highlighted that the regulations exempted only “*chemical or radiation treatments that cause changes in chromosome number or cause chromosome rearrangements*” from regulation as GMOs. Some long-standing *in vitro* chemical treatments do not have these effects, but are caught by this definition. Thus, techniques such as EMS mutagenesis that cause point mutations rather than changes in chromosome number or chromosome rearrangements are regarded technically as GMOs.

In response to these inconsistencies the government held a review of the not genetically modified regulations. The review, which included a public consultation process, resulted in changes intended to maintain the intent of the 1998 regulations and address the drafting errors present in the original regulations. The wording was changed such that mutagenesis techniques that were in use before 1998 were not regulated whilst those developed later are regulated as GMOs. This was done by simply excluding from regulation “*mutagenesis that uses chemical or radiation treatments that were in use on or before 29 July 1998*” (Table 2c) (The New Zealand Government, 2016b). Mutagenesis techniques developed later, including gene editing, however similar they are to the original excluded techniques are regulated as GMOs.

## Future outlook

Gene editing continues to rapidly evolve with developments such as new enzyme capabilities (Yin et al., 2018), base editing (Komor et al., 2016) and simultaneous multi-target approaches, (Svitashev et al., 2015; Chilcoat et al., 2017; Shen et al., 2017) increasing the scope and applicability of the technology. The recent demonstration of rapid *de novo* gene editing-based domestication of wild type relatives of domestic crops without the need for a long breeding programme (Zsögön et al., 2017) has particular applicability in NZ. Particular examples are kiwifruit and radiata pine which are relatively undomesticated and/or where a large number of wildtype genotypes are available (Ferguson, 2007) but require the introduction of essential commercial traits such as longer post-harvest storage and shelf life.

Recent decisions in USA (Waltz, 2018) and the UK (Rogowsky and Wilhelm, 2018) indicate that crops produced using gene editing-based targeted mutagenesis will be able to go to market without going through a time-consuming and burdensome regulatory process required for GMO crops. This regulatory approach will drastically reduce the time to market and compliance costs for gene edited crops. The recent US Department of Agriculture (USDA) approval of *Camelina sativa* edited for enhanced omega-3 oil was completed in 2 years at a much lower cost than the estimated US$30-50 million and 6 years plus that would have been required to fulfill the full USDA process (Waltz, 2018).

In contrast, NZ has adopted a wait-and-see-approach with regard to the regulation of gene editing. The government indicating that a cautious approach is appropriate because as an exporter of billions of dollars of food products we need to be mindful of market perceptions as well as the science (The New Zealand Government, 2016b). It should be noted that the three largest importers of NZ primary products, China, Australia and USA all currently grow GM crops and Australia and China seem likely to follow the lead of USA in not regulating gene edited crops. The current NZ approach prevents rapid implementation of non-transgenic gene editing and also places the extremely high regulatory compliance costs associated with GM research on developers of such technology.

For NZ to maintain its current global competiveness it is essential that industry is able to continue to implement innovative solutions. For this to happen with gene editing, it will be necessary for the government to be proactive in ensuring NZ is in step with global competitors and that innovation is not stifled by the current outdated regulations. Despite the opinion released in January, by the advocate-general of the European Court of Justice, that gene edited crops that did not contain foreign DNA could be exempted from the GMO regulations, the EU has recently decided to adopt a similar regulatory approach to that of NZ. All gene edited crops will be subject to the same stringent regulations as conventional genetically engineered organisms (Callaway, 2018). This makes a global consensus on regulation of gene editing impossible in the immediate future. Although it is too early to judge the long-term impacts of this decision on the global uptake of gene editing or the regulatory approach that will be taken by currently undecided nations, the existence of different regulatory systems will undoubtedly create many challenges, particularly for those nations with strong trading links with the EU.

## Author contributions

All authors listed have made a substantial, direct and intellectual contribution to the work, and approved it for publication.

### Conflict of interest statement

The authors declare that the research was conducted in the absence of any commercial or financial relationships that could be construed as a potential conflict of interest.
